# TNF/TNFR signal transduction pathway-mediated anti-apoptosis and anti-inflammatory effects of sodium ferulate on IL-1β-induced rat osteoarthritis chondrocytes *in vitro*

**DOI:** 10.1186/ar4085

**Published:** 2012-11-07

**Authors:** Jun Qin, Liang Shang, An-song Ping, Jing Li, Xiao-jun Li, Hong Yu, Jacques Magdalou, Liao-bin Chen, Hui Wang

**Affiliations:** 1 Department of Pharmacology, Basic Medical School, Wuhan University, Donghu Road 169, Wuhan 430071, China; 2 Department of Orthopaedic Surgery, Zhongnan Hospital, Wuhan University, Donghu Road 169, Wuhan 430071, China; 3 Department of Biochemistry, Basic Medical School, Wuhan University, Donghu Road 169, Wuhan 430071, China; 4 UMR7561 CNRS-UHP, Laboratoire de Physiopathologie et Pharmacologie Articulaires, Faculté de Médecine, Vandoeuvre-lès-Nancy Cedex, Lorraine 54505, France

## Abstract

**Introduction:**

Sodium ferulate (SF) is a natural component of traditional Chinese herbs. Our previous study shows that SF has a protective effect on osteoarthritis (OA). The objective of this study was to investigate the effect of SF on the TNF/TNF receptor (TNFR) signal transduction pathway of rat OA chondrocytes.

**Methods:**

Primary rat articular chondrocytes were co-treated with IL-1β and SF. Chondrocyte apoptosis was assessed by fluorescein isothiocyanate-annexin V/propidium iodide assay. The PCR array was used to screen the expression of 84 key genes involved in apoptosis. The release of TNFα and prostaglandin E_2 _were analyzed by ELISA. Expressions of proteins were assessed by western blotting. The activity of NF-κB was determined by electrophoretic mobility shift assay (EMSA). Gene expression of inducible nitric oxide synthase (iNOS) was evaluated by real-time quantitative PCR. The nitric oxide content was measured with the Griess method.

**Results:**

After treatment with SF, the apoptosis rate of chondrocytes significantly attenuated (*P *< 0.01). Results of the apoptosis PCR array suggested that mRNA expression of some core proteins in the TNF/TNFR pathway showed valuable regulation. The protein expressions of TNFα, TNFR-1, TNF receptor-associated death domain, caspase-8 and caspase-3 were prevented by SF in a concentration-dependent manner. SF also inhibited activities of caspase-8 and caspase-3 compared with the OA model control (*P *< 0.01). TNF receptor-associated factor-2 expression, phosphorylations of inhibitor of NF-κB kinase (IKK) subunits alpha and beta, and NF-κB inhibitor, alpha (IκBα) were all concentration-dependently suppressed by SF treatment. The results of EMSA showed that SF inhibited the activity of NF-κB. In addition, the expressions of cycloxygenase-2 and iNOS and the contents of prostaglandin E_2 _and NO were attenuated with the treatment of SF (*P *< 0.01).

**Conclusion:**

SF has anti-apoptosis and anti-inflammatory effects on an OA model induced by IL-1β *in vitro*, which were due to inhibitory actions on the caspase-dependent apoptosis pathway and the IKK/NF-κB signal transduction pathway of the TNF/TNFR pathway.

## Introduction

Osteoarthritis (OA) is the most common arthropathy of load-bearing articulating joints in humans and animals. OA is grossly characterized by the degeneration of articular cartilage and the loss of cartilage matrix in affected joints. The pathological process of the disease involves changes in the survival of chondrocytes and is often associated with an inflammatory response. Chondrocytes are the only cells in articular cartilage, which play an important role in maintaining matrix integrity, pathological cascade process and tissue homeostasis. Beyond the compensation capability of chondrocytes in the OA process, apoptosis cells become the main source of various catabolic factors, such as proteases, proinflammatory mediators [1], nitric oxide (NO) and oxygen radicals [2]. Chondrocyte survival or apoptosis and inflammation are therefore important in the pathogenesis of OA.

Over the past decade, apoptosis has been identified as a critical factor responsible for cell loss in ageing OA cartilage. Many important mediators, including IL-1β, TNFα, caspase-8 and caspase-3, are involved in OA chondrocyte apoptosis [3]. IL-1β is one of the main cytokines that has been implicated in the pathogenesis of OA. This cytokine induces large-scale apoptosis in chondrocytes, which leads to further degenerative changes in cartilage [4,5]. Moreover, it has been suggested that IL-1β induces the expression of the TNFα gene in chondrocytes [6] and upregulates the surface expression of TNF receptor (TNFR) [7]. The death receptor, mitochondrial and endoplasmic reticulum pathways are concluded to be the major cellular pathways of apoptosis [8]. These pathways are distinct in initiation and signaling, but a significant overlap exists in regulatory and effector mechanisms. The best known examples of death receptors include Fas and TNFR [4]. Upon binding of the respective ligand, protein interaction modules are used to assemble a receptor signaling complex called the death inducing signaling complex. This complex recruits and activates the upstream initiator caspases, including caspase-8, which leads, in turn, to the activation of effector caspases (caspase-3, caspase-6, caspase-7) and to internucleosomal DNA fragmentation [4].

Inflammation is another promoting factor in the OA process, including chondrocyte and synovium inflammation [9]. The majority of the proinflammatory proteins linked to arthritis are regulated by the transcription factor NF-κB, which regulates the expression of a wide variety of genes [10]. Combination of TNF and TNFR activates TNF receptor-associated factor (TRAF)-2 to lead to the phosphorylation of inhibitor of NF-κB kinase (IKK). The IKK family consists of two catalytic subunits (IKKα and IKKβ) and a noncatalytic regulatory subunit (IKKγ) [11]. The canonical NF-κB signaling (RelA:p50) relies upon IKKγ-IKKβ-mediated degradation of NF-κB inhibitor, alpha (IκBα), and the noncanonical NF-κB signaling (RelB:p52) relies upon NF-κB inducing kinase and IKKα [12,13]. Yong and colleagues showed that IKKβ is the main IKK catalytic subunit responsible for IκBα signal response domain phosphorylation in response to proinflammatory stimuli in chondrocytes [14]. In response to extracellular stimuli, such as TNFα and IL-1β, the transcription factor NF-κB is often activated and subsequently facilitates the transcription of a number of genes involved in inflammation, such as cyclooxygenase-2 (COX-2), inducible nitric oxide synthase (iNOS), and specific cytokines [15]. The induced iNOS catalyzes the formation and release of a large amount of NO, which then plays a key role in OA pathophysiology. Induced by several stimuli, COX-2 is responsible for the production of large amounts of proinflammatory prostaglandins at the inflammatory site [16].

Sodium ferulate (SF), a sodium salt of ferulic acid (3-methoxy-4-hydroxy-cinnamate sodium), is a natural component of traditional Chinese herbs and some foodstuffs. SF can be easily synthesized and is widely used in scientific research and clinical treatment [17-21]. Various beneficial effects of SF have been reported, including as an antioxidant [17], for the removal of free radicals [18,19], anti-inflammatory actions [20] and anti-apoptotic activity [21]. Recently, for the first time our laboratory showed the beneficial effect of SF on OA [22]. We showed that SF reverses cartilage degradation processes and inhibits expressions of matrix metalloproteinase-1 and BAX in a rat OA model *in vivo*. We also showed that SF prevents chondrocytes apoptosis, represses NO synthesis and attenuates the levels of matrix metalloproteinase-1/tissue inhibitor of metalloproteinase-1 to prevent extracellular matrix degradation in human OA chondrocytes [22]. However, the precise molecular mechanism responsible for SF's anti-apoptosis and anti-inflammatory effects in chondrocytes is not yet clear. According to the screen results of an apoptosis RT^2 ^profile PCR array, the aim of the present study was to investigate the effect of SF on the TNF/TNFR signal transduction pathway, including the caspase-dependent apoptosis pathway and the NF-κB signal transduction pathway, in rat OA chondrocytes.

## Materials and methods

### Materials

SF was provided by YaoYou Medicine (Chongqing, China; 0.1 g/ampere, SFDA approval number H50021634). IL-1β was purchased from PeproTech (Rocky Hill, NJ, USA). Annexin V conjugated to fluorescein isothiocyanate (annexin-V-FITC) and propidium iodide (PI) were from Jingmei Biotech (Wuhan, China). The Rat Apoptosis RT^2 ^Profiler PCR array PARN-012 and the RT^2 ^First Strand kit were purchased from SABiosciences (Frederick, MD, USA). TNFα and prostaglandin E_2 _(PGE2) ELISA kits were obtained from R&D Systems (Minneapolis, MN, USA). Rabbit anti-TNFR-1 polyclonal antibody, anti-TNF receptor-associated death domain (anti-TRADD), anti-TRAF-2, anti-IKKα, anti-phospho-IKKα, anti-IKKβ, anti-phospho-IKKβ, anti-IκBα, anti-phospho-IκBα, anti-caspase-3, anti-COX-2, anti-glyceraldehyde-3-phosphate dehydrogenase (GAPDH) and horseradish peroxidase-conjugated anti-rabbit were obtained from Santa Cruz Biotechnology (Santa Cruz, CA, USA). Rabbit anti-caspase-8 was purchased from BoAoSen Biotech (Wuhan, China). The Membranous, Nuclear and Cytoplasmic Protein Extraction Kit, the BCA Protein Assay Kit and the caspase-3, caspase-8 Activity Assay Kit were obtained from Beyotime Biotech (Shanghai, China). The enhanced chemiluminescence kit and biotin-labeled double-stranded oligonucleotide probes were provided by Pierce Biotech (Rockford, IL, USA) and Viagen Biotech (Los Angeles, CA, USA), respectively. All other reagents were sourced from Sigma-Aldrich (Saint Louis, MO, USA), unless otherwise indicated.

### Chondrocyte culture and treatment

Animal care and treatment were in accordance with the Guidelines of the Laboratory Animal Management and Review Committee of Wuhan University (China). Male Wistar rats (130 to 150 g, Animal Center of Wuhan University) were housed under controlled temperature and lighting conditions with food and water. Articular cartilage isolated from femoral head cap pieces was aseptically dissected, and chondrocytes were obtained after digestion of cartilage fragments in 0.25% trypsin (w/v) for 30 minutes followed by about 6 to 7 hours of digestion in 0.2% collagenase II (w/v) in DMEM without serum. Chondrocytes were cultured at a density of 10^5 ^cells/ml in DMEM with 10% fetal bovine serum. Experiments were performed with first-passage cultures. SF was prepared with sterile distilled water and diluted with DMEM. The concentration of SF was selected on the basis of our previous study [22]. Chondrocytes were divided into six groups: normal control group, chondrocytes without any treatments; OA model control group, chondrocytes treated with 20 ng/ml IL-1β; and SF treatment groups, OA model chondrocytes treated with 20 ng/ml IL-1β and 125, 250, 500 or 1,000 μmol/l SF, respectively. Chondrocytes were serum starved and co-treated with 125, 250, 500 or 1,000 μmol/l SF and 20 ng/ml IL-1β for 48 hours after incubation with the different concentrations SF mentioned above alone for 24 hours. To investigate whether different concentrations of SF themselves present cytotoxicity on chondrocyte (detection of cell viability), normal chondrocytes were treated with 125, 250, 500 or 1,000 μmol/l SF, respectively, for 72 hours, followed by an assay of cell viability. These experiments were performed in triplicate and the results are provided as mean values from three independent experiments.

### Chondrocyte viability assay

After chondrocytes had been cultured in 96-well flasks (10^4 ^cells/well) for 48 hours, 125, 250, 500 or 1,000 μmol/l SF were added for 72 hours, respectively. The medium was then replaced with DMEM containing 5 mg/ml 3-(4,5-dimethyl-2-thiazolyl)-2,5-diphenyl-2H-tetrazolium bromide and incubated for 4 hours at 37°C. Formazan products were dissolved in 100 μl dimethylsulfoxide and absorbance was measured at 570 nm using a microplate reader (Shimadzu, Kyoto, Japan).

### Detection of apoptosis

Chondrocytes were resuspended in 200 μl HEPES buffer, and stained with 5 μl annexin-V-FITC and 10 μl PI for 15 minutes at room temperature in the dark. After incubation, 200 μl HEPES buffer was added, and the cells were measured by EPICS ALTRAII flow cytometry (Beckman, Fullerton, CA, USA) and analyzed with Multi-cycle software (Phoenix Flow Systems, San Diego, CA, USA). Results were expressed as the percentage of (PI-negative and annexin-V-positive) apoptotic cells. All experiments were performed in triplicate.

### Chondrocyte apoptosis PCR array and real-time quantitative PCR

Total RNAs were extracted from chondrocytes using TRIzol (Invitrogen, Carlsbad, CA, USA) following the manufacturer's instructions. RNA concentrations were measured using the Nanodrop method (3300 NanoDrop Analyzer; Thermo Scientific, Wilmington, NC, USA). Equal amounts of RNA per sample (1 μg) were used for the RT^2 ^First Strand kit. Comparison of the relative expression of 84 apoptosis-related genes was performed using the RT^2 ^Profiler PCR array PARN-012 (Rat Apoptosis PCR Array; SABiosciences) on an ABI Fast 7500 thermocycler using RT2 Real-Time SYBR Green PCR master mix PA-012 (SABiosciences, Frederick, MD, USA). Hypoxanthine phosphoribosyltransferase-1, GAPDH, and β-actin (ACTB) housekeeping genes were used for normalization and data were analyzed with the ΔΔCt (threshold cycle) method. The real-time PCR primers for GAPDH and iNOS were designed corresponding to the coding region of the genes as follows: GAPDH, sense 5'-GGCTCTCTGCTCCTCCCTGT-3' and antisense 5'-GTAACCAGGCGTCCGATACGGC-3'; iNOS, sense 5'-TTCTGTGCTAATGCGGAAGGT-3' and antisense 5'-GCTTCCGACTTTCCTGTCTCA-3'. After an initial incubation for 15 minutes at 95°C, the reactions were carried out for 40 cycles at 95°C for 15 seconds and 60°C for 30 seconds (florescence collection). The expression of iNOS was normalized to the GAPDH gene to standardize comparison.

### Enzyme-linked immunosorbent assay

Chondrocytes were cultured and stimulated as described above and supernatants were collected after 72 hours. The release of TNFα and PGE2 were analyzed by ELISA kits (R&D Systems, Minneapolis, MN, USA) according to the manufacturer's instructions. The optical density of each well was determined within 30 minutes, using a microplate reader set to 450 nm. TNFα and PGE2 values are expressed as picograms per milliliter.

### Western blotting analysis

Chondrocyte monolayers were washed three times with cold PBS and cell proteins were extracted on ice by the Membranous, Nuclear and Cytoplasmic Protein Extraction Kit with 1 mmol/l phenylmethyl sulfonylfluoride. The protein concentration of different extracts was determined according to the BCA Protein Assay Kit using BSA as a standard. After adjusting to equal amounts (50 μg protein per lane) of proteins, they were separated on SDS-PAGE (5 and 12% gels) under reducing conditions and transferred to polyvinylidene difluoride membranes. Membranes were blocked and probed with anti-rat TNFR-1 (1:200), TRAF-2 (1:400), IKKα (1:300), phospho-IKKα (1:300), IKKβ (1:300), phospho-IKKβ (1:300), IκBα (1:300), phospho-IκBα (1:300), COX-2 (1:400), GAPDH (1:300), anti-rat caspase-8 (1:200), caspase-3 (1:200) or TRADD (1:200) primary antibodies, then incubated with horseradish peroxidase-conjugated secondary antibodies (goat anti-rabbit IgG, 1:3,000), followed with visualization by the enhanced chemiluminescence kit.

### Caspase protease activity assay

Caspase activity was determined by a colorimetric assay based on the ability of caspase-3 and caspase-8 to change acetyl-Asp-Glu-Val-Asp *p*-nitroanilide (Ac-DEVD-*p*NA) and acetyl-Ile-Glu-Thr-Asp *p*-nitroanilide (Ac-IETD-*p*NA) into a yellow formazan product of *p*-nitroaniline, respectively. An increase in absorbance at 405 nm was used to quantify the activation of caspase activity. The collected chondrocytes were rinsed with cold PBS, and then lysed by lysis buffer (40 μl) for 15 minutes on ice. Cell lysates were centrifuged at 16,000 × *g *for 10 minutes at 4°C. Caspase-3 and caspase-8 activities in the supernatant were assayed with the caspase-3, caspase-8 Activity Assay Kit. An activity unit was defined as the amount of enzyme that will cleave 1.0 nmol colorimetric substrate Ac-DEVD-*p*NA or Ac-IETD-pNA per hour at 37°C under saturated substrate concentrations. Total protein contents were determined according to the BCA Protein Assay Kit and the caspase activity was expressed as activity units compared with total protein content (unit/μg protein). All of the experiments were carried out in triplicate.

### NF-κB activity assay

Nuclear extracts (5 mg per reaction) were incubated with biotin-labeled double-stranded oligonucleotide probes (P1, 5'-AGTTGAGGGGACTTTCCCAGGC-3'; P2, 3'-TCAACT CCCCTGAAAGGGTCCG-5'; Viagen Biotech) for 20 minutes at room temperature. Reactions were fractionated on nonreducing 6.5% polyacrylamide gel and transferred to nylon membranes, then incubated with streptonuclide-horseradish peroxidase and visualized.

### Nitrite assay

NO production was measured as nitrite (NO_2_^-^) in the culture medium. As reported previously [22], nitrite was detected by the Griess reaction using sodium nitrite as the standard. Absorbance at 550 nm was measured on a UV-1601 spectrophotometer (Shimadzu, Kyoto, Japan). The nitrite concentration was calculated and expressed in micromoles per liter.

### Statistical analysis

Results are presented as the mean ± standard error of the mean. After testing for normal distribution (Kolmogorov-Smirinov), a paired Student's *t *test was used to compare data between groups. To analyze the effects of treatments, two-way analysis of variance for repeated measures with appropriate *post hoc *comparisons (Student-Newman-Keuls) was performed. *P *< 0.05 was considered significant.

## Results

### Assay of cell viability

**Figure 1:**
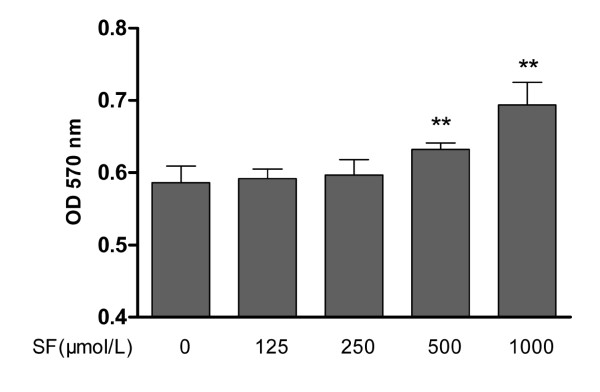
**Effect of sodium ferulate on proliferation of normal rat chondrocytes**. After chondrocytes had been cultured in a 96-well plate for 48 hours, 125, 250, 500 or 1,000 μmol/l sodium ferulate (SF) were added for 72 hours, respectively. Values represent mean ± standard error of the mean of eight different wells. **P < 0.01 versus control (0). OD, optical density.

Monolayer cultured chondrocyte viability in the SF group was significantly higher than in the control group at 500 and 1,000 μmol/l dosages (*P *< 0.01; *n *= 8). However, 125 and 250 μmol/l SF had no effect on increasing cell viability. All of these results show that different concentrations of SF from 125 to 1,000 μmol/l have no effect on normal chondrocyte viability (Figure 1).

### Chondrocyte apoptosis

**Figure 2:**
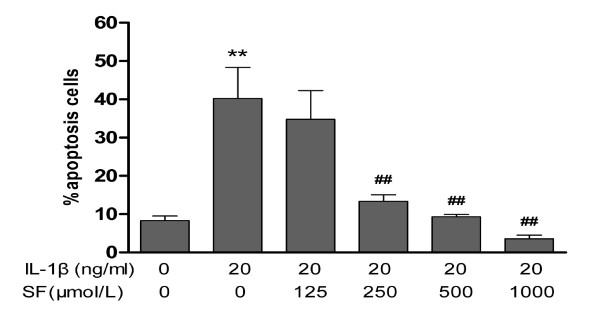
**Effect of sodium ferulate on the apoptosis rate of IL-1β-induced rat osteoarthritis chondrocytes**. Chondrocytes were incubated with sodium ferulate (SF) alone for 24 hours, and then co-treated with IL-1β and SF for 48 hours. After staining with annexin-V-fluorescein isothiocyanate and propidium iodide, chondrocytes were measured on flow cytometry and analyzed with Multi-cycle software (Phoenix Flow Systems, San Diego, CA, USA). Results expressed as percentage of apoptotic cells. Values represent mean ± standard error of the mean of four different simples. **P < 0.01 versus normal control. ^##^*P *< 0.01 versus osteoarthritis model control.

Chondrocyte apoptosis was identified by FITC-annexin V/PI double-labeled assay. IL-1β significantly increased the percentage of apoptotic chondrocytes in model control compared with the normal control (*P *< 0.01; *n *= 4). With treatments of 250, 500 and 1,000 μmol/l SF, the apoptotic percentage of OA chondrocytes was attenuated in a concentration-dependent manner (all *P *< 0.01; *n *= 4). However, 125 μmol/l SF failed to prevent OA chondrocyte apoptosis (Figure 2).

### Chondrocyte apoptosis PCR array

The Rat Apoptosis RT^2 ^Profiler PCR array analysis was performed for expression of 84 apoptosis genes in the normal control, model control and 1,000 μmol/l SF treatment groups. The array included TNF ligands and their receptors (TRAFs), Bcl2 family members, caspases, inhibitor of apoptosis, caspase recruitment domain family members, death domain, death effector domain, and cell death-inducing DFFA-like effector family members, as well as the genes involved in the p53 and DNA-damaged induced apoptosis and ataxia telangiectasia mutated pathways. Each reported value represents the mean fold of mRNA expression relative to the control levels for three biological replicates. To focus on particular pathways, a cutoff value for the fold-change ≥2.0 was taken. The mRNA expression of TNF increased 30.07-fold after stimulation by IL-1β. The mRNA expressions of TNFR-1B, TRAF-2, TRADD, Fas-associated death domain protein, caspase-8, caspase-3 and NF-κB increased respectively after stimulation by IL-1β versus normal control. However, SF could reduce the mRNA expressions of these core proteins respectively after treatment with SF versus the OA model control (Table 1).

**Table 1:** Effects of 1,000 μmol/l sodium ferulate on apoptosis-related gene expression in rat osteoarthritis chondrocytes.

Gene	Model control/normal control (20 ng/ml vs. 0 ng/ml IL-1β)	SF treatment/model control (1,000 μmol/l vs. 0 μmol/l SF in presence of 20 ng/ml IL-1β)
TNF/TNFR pathway		
TNF	30.07	0.64
TNFR-1b	3.04	0.39
TRAF-2	2.50	0.43
TRADD	1.98	0.69
Caspase-8	2.30	0.43
Caspase-3	1.88	0.46
NF-κB	2.55	0.56
Other pathways		
Fas	1.85	1.00
Fas ligand	0.45	0.42
FADD	0.94	0.58
Bcl-2	0.75	0.59
Bax	0.61	0.66
Bax/Bcl-2	0.80	1.12
Apaf1	0.76	1.03
Caspase 9	0.53	0.48

Each reported value represents the mean fold of mRNA expression relative to the control levels for three biological replicates. FADD, Fas-associated death domain protein; SF, sodium ferulate; TNFR, tumor necrosis factor receptor; TRADD, TNF receptor-associated death domain; TRAF, TNF receptor-associated factor.

### Caspase cascade apoptosis pathway induced by TNF/TNF receptor

**Figure 3:**
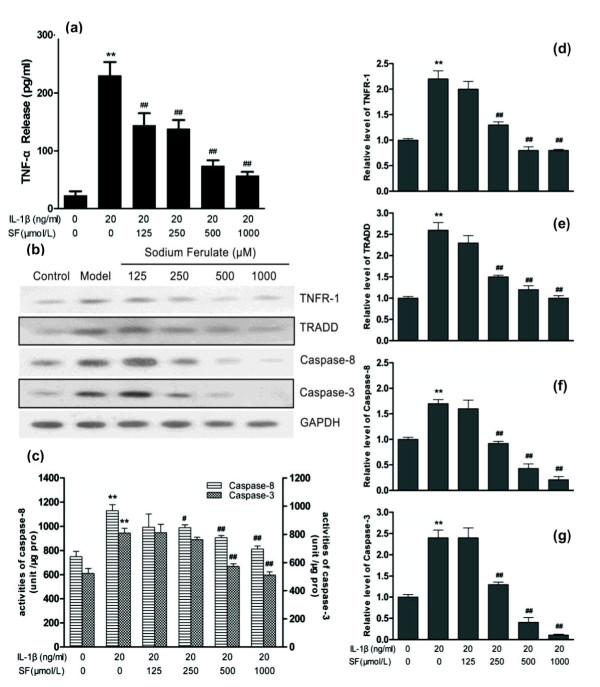
**Effects of sodium ferulate on the caspase cascade apoptosis pathway in rat osteoarthritis chondrocytes**. Effects of sodium ferulate (SF) on the TNF/TNF receptor (TNFR)-mediated caspase cascade apoptosis pathway of rat osteoarthritis (OA) chondrocytes induced by IL-1β. **(a) **Effects of SF on expression of TNFα in rat OA chondrocytes. Chondrocytes were incubated with SF alone for 24 hours, and then co-treated with IL-1β and SF for 48 hours. Supernatants were collected after 72 hours. Release of TNFα was analyzed by ELISA. **(b) **Effects of SF on protein expression of TNFR-1, TNF receptor-associated death domain (TRADD), caspase-8, caspase-3 and glyceraldehyde-3-phosphate dehydrogenase (GAPDH) in rat OA chondrocytes. Expression of proteins determined by western blotting. **(c) **Effects of SF on activity of caspase-8 and caspase-3 in rat OA chondrocytes. An activity unit was defined as the amount of enzyme that will cleave 1.0 nmol colorimetric substrate (acetyl-Asp-Glu-Val-Asp *p*-nitroanilide or acetyl-Ile-Glu-Thr-Asp *p*-nitroanilide) per hour at 37°C under saturated substrate concentrations. Caspase activity was expressed as activity units compared with total protein content (unit/μg pro). **(d, e, f, g) **Relative level of TNFR-1, TRADD, caspase-8 and caspase-3 normalized to GAPDH and compared with normal control, quantitatively analyzed by Kodak Digital Science 1D software (Eastman Kodak, Rochester, NY, USA) and expressed as mean optical density. Values represent mean ± standard error of the mean of three different simples. **P < 0.01 versus normal control. ^#^*P *< 0.05, ^##^*P *< 0.01 versus OA model control.

ELISA showed that IL-1β induced the large-scale release of TNFα (*P *< 0.01; *n *= 3). Western blotting clearly showed a similar GAPDH content in each group and an increase in TNFR-1, TRADD, caspase-8 and caspase-3 expression contents in the model control compared with normal control. Treatment with 250, 500 and 1,000 μmol/l SF can obviously inhibit each protein expression induced by IL-1β in a concentration-dependent manner, especially on caspase-8 and caspase-3 (all *P *< 0.01; *n *= 3) (Figure 3a,b).

IL-1β significantly increased activities of caspase-8 and caspase-3 in the model group (both *P *< 0.01; *n *= 3). However, SF concentration-dependently attenuated the activities of caspase-8 and caspase-3 in OA chondrocytes at 250 to 1,000 μmol/l, except for 250 μmol/l SF on caspase-3 (all *P *< 0.01 or 0.05; *n *= 3). Treatment with 125 μmol/l SF had no effect on activities of caspase-8 and caspase-3 in OA chondrocytes (Figure 3c).

### IKK/NF-κB pathway induced by TNF/TNF receptor

**Figure 4:**
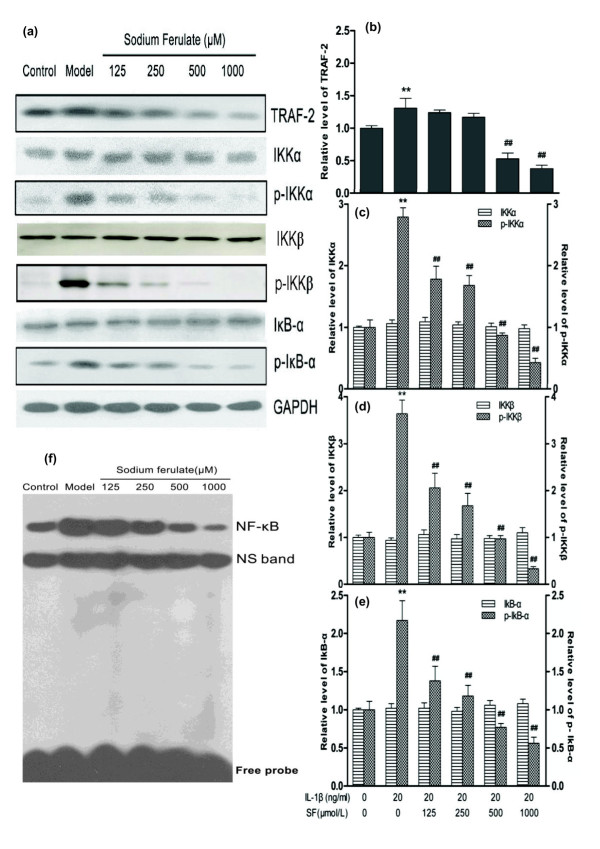
**Effects of sodium ferulate on the IKK/NF-κB signaling pathway of rat osteoarthritis chondrocytes**. Effects of sodium ferulate (SF) on the TNF/TNF receptor (TNFR)-mediated IKK/NF-κB signaling pathway of rat osteoarthritis (OA) chondrocytes induced by IL-1β. **(a) **Effects of SF on protein expression of TNF receptor-associated factor 2 (TRAF-2), inhibitor of NF-κB kinase (IKK) subunit alpha (IKKα), IKK subunit beta (IKKβ), NF-κB light polypeptide gene enhancer in B-cell inhibitor, alpha (IκBα), phosphorylation of IKKα (p-IKKα), phosphorylation of IKKβ (p-IKKβ), phosphorylation of IκBα (p-IκBα) and glyceraldehyde-3-phosphate dehydrogenase (GAPDH) in rat OA chondrocytes. Chondrocytes were incubated with SF alone for 24 hours, and then co-treated with IL-1β and SF for 48 hours. Expressions of proteins determined by western blotting. **(b, c, d, e) **Relative level of TRAF-2, IKKα, IKKβ, IκBα, p-IKKα, p-IKKβ and p-IκBα normalized to GAPDH and compared with normal control, quantitatively analyzed by Kodak Digital Science 1D software (Eastman Kodak, Rochester, NY, USA) and expressed as mean optical density. Values represent mean ± standard error of the mean of three different simples. **P < 0.01 versus normal control. ^##^*P *< 0.01 versus OA model control. **(f) **Effects of SF on activity of NF-κB in rat OA chondrocytes. Nuclear extracts were incubated with biotin-labeled double-stranded oligonucleotide probes. Reactions were fractionated on nonreducing 6.5% polyacrylamide gel and transferred to nylon membranes, then incubated with streptonuclide-horseradish peroxidase and visualized. NS, nonspecific free DNA band.

Expressions of protein TRAF-2, phospho-IKKα, phospho-IKKβ and phospho-IκBα were induced by IL-1β, but IKKα, IKKβ and IκBα expressions were similar with the normal control. Concentration-dependent attenuations in TRAF-2, phospho-IKKα, phospho-IKKβ and phospho-IκBα protein expressions were markedly present after treatment of 500 and 1,000 μmol/l SF (all *P *< 0.01; *n *= 3), but IKKα, IKKβ and IκBα protein expressions showed no obvious alteration (*n *= 3). The GAPDH content was similar in each lane (Figure 4a,b,c,d,e).

EMSA results showed that IL-1β markedly increased the binding activity of NF-κB to DNA, but SF inhibited this activity in a concentration-dependent manner compared with the model control (Figure 4f).

**Figure 5:**
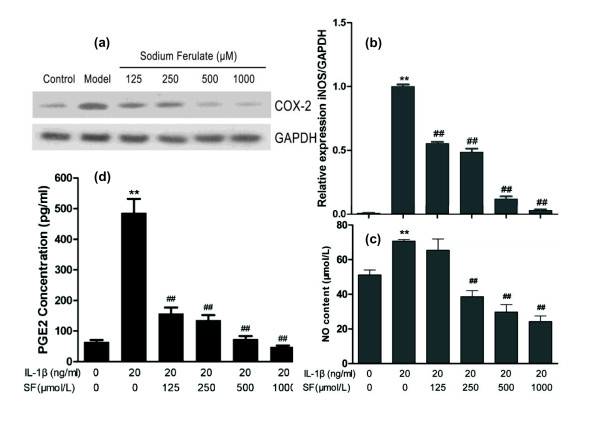
**Effects of sodium ferulate on cycloxygenase-2, prostaglandin E2, inducible nitric oxide synthase, and nitric oxide**. Effects of sodium ferulate (SF) on cycloxygenase-2 (COX-2), prostaglandin E2 (PGE2), inducible nitric oxide synthase (iNOS) and nitric oxide (NO) expression and synthesis of rat osteoarthritis (OA) chondrocytes induced by IL-1β. **(a) **Effects of SF on protein expression of COX-2 and glyceraldehyde-3-phosphate dehydrogenase (GAPDH) in rat OA chondrocytes. Chondrocytes were incubated with SF alone for 24 hours, and then co-treated with IL-1β and SF for 48 hours. Expression of COX-2 and GAPDH determined by western blotting. **(b) **Effects of SF on gene expression of iNOS and GAPDH in rat OA chondrocytes. Level of iNOS was evaluated by real-time quantitative PCR. **(c) **Effects of SF on NO synthesis of rat OA chondrocytes induced by IL-1β. NO production was measured as nitrite (NO_2_^-^) in the culture medium by the Griess reaction. NO concentration was calculated (expressed in μmol/l). **(d) **Effects of SF on expression of PGE2 in rat OA chondrocytes. The release of PGE2 was analyzed by ELISA and the concentration was calculated (expressed in pg/ml). Values represent mean ± standard error of the mean of three different simples. **P < 0.01 versus normal control. ^##^*P *< 0.01 versus OA model control.

Expressions of COX-2 and PGE2 induced by IL-1β increased in the model control (both *P *< 0.01, *n *= 3). SF concentration-dependently attenuated COX-2 expression and PGE2 release (all *P *< 0.01, *n *= 3). IL-1β significantly increased the gene expression of iNOS and NO synthesis in the model group (both *P *< 0.01; *n *= 3). However, SF concentration-dependently inhibited the gene expression of iNOS and NO synthesis with the exception of the 125 μmol/l SF treatment group in NO content (Figure 5a,b,c,d).

## Discussion

IL-1β is a proinflammatory cytokine in OA pathogenesis, which is widely used on chondrocytes to establish an OA model *in vitro *[23,24]. Pathologic effects of IL-1β include: inhibition of chondrocyte proliferation and synthesis of collagen II and proteoglycan in the extracellular matrix [25]; increase of cartilage catabolic enzyme activity, such as matrix metalloproteinases, which lead to degradation of extracellular matrix [26]; promotion of NO, free radical and inflammatory factor synthesis [27]; and activation of some intracellular key factors on the signal pathway, such as NF-κB [28]. When NF-κB is activated by IL-1β, gene expressions of TNFα, iNOS and COX-2 obviously increase [28-31]. Further research showed that NF-κB reached the activity peak at 30 minutes after treatment of IL-1β, and then attenuated to an undetectable level [32]. Notably, at this time, OA progress was still aggravated because of abundant factors, such as TNFα, iNOS and COX-2, induced by activated NF-κB. Moreover, it has been confirmed that IL-1β might upregulate surface expression of TNFR [7]. Such autocrine and paracrine loops perpetuate joint destruction, frequently resulting in irreversible disease progression. IL-1β can also induce chondrocyte apoptosis to establish an experimental apoptosis model [33]. Our previous findings corroborated the abovementioned effects of IL-1β in primary human OA chondrocytes [22]. SF prevented these sequential effects, but the precise mechanism responsible for its anti-apoptosis and anti-inflammatory effects is not yet clear. In the present study, taking into account the exaggerative increase of TNF gene expression (30.07-fold) in the apoptosis PCR array after treatment of IL-1β for 48 hours, we concluded that abundant TNFα induced by IL-1β initiated the TNF/TNFR signal pathway for chondrocyte apoptosis and inflammation at that time point. The ELISA results also confirmed that IL-1β could induce protein expression of TNFα in chondrocytes.

**Figure 6:**
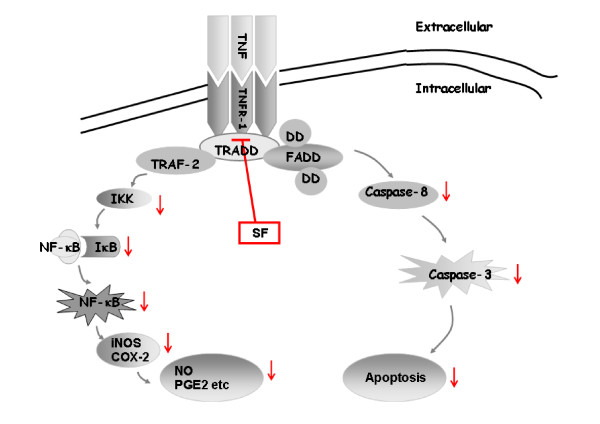
**Effect of sodium ferulate on caspase cascade apoptosis and IKK/NF-κB pathways induced by TNF/TNF receptor**. Combination of TNF and TNF receptor (TNFR) on the cell membrane will activate two downstream signal pathways. One is the caspase cascade apoptosis pathway, which recruits TNF receptor-associated death domain (TRADD), Fas-associated death domain protein (FADD) and death domain (DD) and forms death inducing signaling complex (DISC), the initiator caspases (caspase-8), and activates executioner caspases (caspase-3). Active executioner caspases cleave the death substrates, eventually resulting in apoptosis. The other is the inhibitor of NF-κB kinase (IKK)/NF-κB pathway, which activates the cytoplasmic IKK. Phosphorylated IKK (p-IKK) activates NF-κB inhibitor, alpha (IκBα) into the phosphorylation phase. Phosphorylated IκBα is then ubiquitinated and degraded by the proteasome and active NF-κB is released. NF-κB translocates to the nucleus, where it activates proinflammatory and pro-apoptotic gene production. In rat OA chondrocytes, sodium ferulate (SF) inhibited both the caspase cascade apoptosis pathway and the IKK/NF-κB pathway induced by combination of TNF/TNFR. COX-2, cyclooxygenase-2; NO, nitric oxide; PGE2, prostaglandin E_2_; TRAF, TNF receptor-associated factor.

Combination of TNF and TNFR on the cell membrane can activate the downstream signal pathway. Research shows that a combination of TNF/TNFR induces two signal pathways: the caspase cascade apoptosis pathway and the IKK/NF-κB pathway [34] (Figure 6). Oligomerization of TNF/TNFR by their ligands induces recruitment of adaptor proteins such as Fas-associated death domain protein and TRADD. These adaptor proteins bind to the cytoplasmic tail of receptors through homologous death domain interactions. Procaspase-8 is recruited into the complex via homologous death effector domain interactions, forming death inducing signaling complex. The initiator caspases activate executioner caspases. Active executioner caspases (caspase-3) cleave the death substrates, which eventually results in apoptosis [35]. In the present study, SF concentration-dependently prevented the percentage of apoptotic chondrocytes induced by IL-1β. The screening results from the RT² Profiler Apoptosis PCR Array showed that SF attenuated the gene expressions of TNFR-1, TRADD, caspase-8 and caspase-3, which were related to the TNF/TNFR-induced caspase cascade apoptosis pathway. The results of western blotting and ELISA showed a similar alteration tendency on these proteins, in which SF attenuated the expressions of TNFα, TNFR-1, TRADD, caspase-8 and caspase-3 proteins and the activities of caspase-8 and caspase-3. We speculate that the TNF/TNFR-induced caspase cascade apoptosis pathway may be the molecular mechanism involved in the anti-apoptosis effect of SF on OA.

In addition, recruitment of TRADD after combination of TNF/TNFR activates TRAF-2 to lead to the phosphorylation of IKK. The canonical NF-κB signaling (RelA:p50) relies upon IKKγ-IKKβ-mediated degradation of IκBα, and the noncanonical NF-κB signaling (RelB:p52) relies upon NF-κB inducing kinase and IKKα [12,13]. Recent analyses revealed that synthesis of the constituents of the noncanonical pathway is controlled by canonical IKKβ-IκB-RelA:p50 signaling [36]. Moreover, generation of the canonical and noncanonical dimers within the cellular milieu is also mechanistically interlinked [36]. A broad range of stimuli can activate the NF-κB dimer by triggering a signaling pathway that leads to the phosphorylation of IKK. Activated NF-κB translocates into the nucleus and binds with specific genes involved in inflammation for regulation of transcription [26], such as TNFα, iNOS and COX-2 [37,38]. In addition to the alternation on the caspase cascade apoptosis pathway, the PCR array showed that the executing factor NF-κB of the IKK/NF-κB pathway was attenuated by SF. We therefore determined the relative proteins in the TNF/TNFR-induced IKK/NF-κB pathway and inflammatory factors downstream. These results showed that the expressions of protein TRAF-2, phospho-IKKα, phospho-IKKβ and phospho-IκBα were markedly prevented in a concentration-dependent manner after treatment of SF in chondrocytes induced by IL-1β. After IκBα is rapidly phosphorylated it is ubiquitinated and then subsequently degraded by the proteosome within the next 1 or 2 hours at most [39]. IκBα-NF-κB signaling has multiple interacting negative feedback loops and an autoregulatory pathway [39,40]. In the present model, we found that total IκBα protein levels did not change after IL-1β treatment of 24 to 48 hours. Perhaps this is due to continuous stimulation by IL-1β. Further studies are needed to elucidate the precise mechanism of SF on IκBα at the early stage in OA chondrocytes induced by IL-1β. What is more, our EMSA experiment showed that SF inhibited the binding activity of NF-κB to DNA compared with the model control but could not define precise NF-κB subunits. In the model control group, iNOS gene expression and COX-2 protein expression obviously were increased with intervention of IL-1β. However, SF prevented the increases of iNOS gene expression and COX-2 protein expression in the model group. The contents of NO and PGE2 were also attenuated with the treatment of SF. Combined with changes of relative proteins (such as TNFα, TNFR-1 and TRAF-2), the binding activity of NF-κB and expressions of downstream inflammatory genes (such as COX-2, iNOS, PGE2 and NO), we speculate that SF can attenuate the TNF/TNFR-induced inflammation of OA chondrocytes, which might be due to the suppression of the IKK/NF-κB signaling pathway.

Recently, SF has been approved by State Drugs Administration of China as a drug for treatment of cardiovascular and cerebrovascular diseases [41,42], diabetic nephropathy [43] and neurodegenerative disorders [44]. The safety and efficacy of SF have been demonstrated in clinical practice. Our previous study showed that intraarticular administration of SF can reduce cartilage degradation processes in a rat OA model *in vivo *[22]. The present research revealed the precise molecule mechanism responsible for the anti-apoptosis and anti-inflammatory effects of SF in chondrocytes. All these observations provide the scientific basis for the clinical application of SF on OA.

## Conclusion

Our findings indicate that SF can prevent apoptosis of chondrocytes *in vitro*, due to the inhibitory action of the caspase-dependent pathway of the TNF/TNFR signal transduction pathway. SF can also attenuate the TNF/TNFR-induced inflammation of OA chondrocytes and downregulate the NO synthesis and PGE2 release, which might be due to suppression of the IKK/NF-κB signaling pathway. From our study, the effective protection of SF in OA chondrocytes *in vitro *is involved not only in the anti-apoptosis process but also in the anti-inflammatory effect.

## Abbreviations

Ac-DEVD-*p*NA: acetyl-Asp-Glu-Val-Asp *p*-nitroanilide; Ac-IETD-*p*NA: acetyl-Ile-Glu-Thr-Asp *p*-nitroanilide; BSA: bovine serum albumin; COX-2: cycloxygenase-2; DMEM: Dulbecco's modified Eagle's medium; ELISA: enzyme-linked immunosorbent assay; EMSA: electrophoretic mobility shift assay; FITC: fluorescein isothiocyanate; GAPDH: glyceraldehyde-3-phosphate dehydrogenase; IκBα: NF-κB inhibitor: alpha; IKK: inhibitor of NF-κB kinase; IL: interleukin; iNOS: inducible nitric oxide synthase; NF: nuclear factor; NO: nitric oxide; OA: osteoarthritis; PBS: phosphate-buffered saline; PCR: polymerase chain reaction; PI: propidium iodide; PGE2: prostaglandin E_2_; SF: sodium ferulate; TNF: tumor necrosis factor; TNFR: tumor necrosis factor receptor; TRADD: TNF receptor-associated death domain; TRAF: TNF receptor-associated factor.

## Competing interests

The authors declare that they have no competing interests.

## Authors' contributions

JQ and LS carried out the experimental work, collection, interpretation and manuscript drafting. A-SP and X-JL designed the experiments for this study and drafted the manuscript. JL carried out the experimental work. HY and JM interpreted the data. HW and L-BC designed the experiments for this study, interpreted the data and drafted the manuscript. All authors read, edited and approved the final manuscript.

## References

[B1] Mazzetti I Grigolo B Pulsatelli L Dolzani P Silvestri T Roseti L Meliconi R Facchini A Differential roles of nitric oxide and oxygen radicals in chondrocytes affected by osteoarthritis and rheumatoid arthritis Clin Sci (Lond) 2001 14 593 599 10.1042/CS20010030 11724645

[B2] Venkatesan N Barre L Benani A Netter P Magdalou J Fournel-Gigleux S Ouzzine M Stimulation of proteoglycan synthesis by glucuronosyltransferase-I gene delivery: a strategy to promote cartilage repair Proc Natl Acad Sci USA 2004 14 18087 18092 10.1073/pnas.0404504102 15601778 PMC535800

[B3] Pelletier JP Jovanovic DV Lascau-Coman V Fernandes JC Manning PT Connor JR Currie MG Martel-Pelletier J Selective inhibition of inducible nitric oxide synthase reduces progression of experimental osteoarthritis in vivo: possible link with the reduction in chondrocyte apoptosis and caspase 3 level Arthritis Rheum 2000 14 1290 1299 10.1002/1529-0131(200006)43:6<1290::AID-ANR11>3.0.CO;2-R 10857787

[B4] Csaki C Mobasheri A Shakibaei M Synergistic chondroprotective effects of curcumin and resveratrol in human articular chondrocytes: inhibition of IL-1β-induced NF-κB-mediated inflammation and apoptosis Arthritis Res Ther 2009 14 R165 10.1186/ar2850 19889203 PMC3003513

[B5] Montaseri A Busch F Mobasheri A Buhrmann C Aldinger C Rad JS Shakibaei M IGF-1 and PDGF-bb suppress IL-1β-induced cartilage degradation through down-regulation of NF-κB signaling: involvement of Src/PI-3K/AKT pathway PLoS One 2011 14 e28663 10.1371/journal.pone.0028663 22194879 PMC3237481

[B6] Campo GM Avenoso A D'Ascola A Scuruchi M Prestipino V Calatroni A Campo S Hyaluronan in part mediates IL-1β-induced inflammation in mouse chondrocytes by up-regulating CD44 receptors Gene 2012 14 24 35 10.1016/j.gene.2011.11.064 22192912

[B7] Saperstein S Chen L Oakes D Pryhuber G Finkelstein J IL-1β augments TNF-α-mediated inflammatory responses from lung epithelial cells J Interferon Cytokine Res 2009 14 273 284 10.1089/jir.2008.0076 19231998 PMC2718541

[B8] Kim HA Blanco FJ Cell death and apoptosis in osteoarthritic cartilage Curr Drug Targets 2007 14 333 345 10.2174/138945007779940025 17305511

[B9] Akhtar N Haqqi TM Epigallocatechin-3-gallate suppresses the global interleukin-1β-induced inflammatory response in human chondrocytes Arthritis Res Ther 2011 14 R93 10.1186/ar3368 21682898 PMC3218908

[B10] Venkatesha SH Berman BM Moudgil KD Herbal medicinal products target defined biochemical and molecular mediators of inflammatory autoimmune arthritis Bioorg Med Chem 2011 14 21 29 10.1016/j.bmc.2010.10.053 21115252 PMC3020797

[B11] Li X Massa PE Hanidu A Peet GW Aro P Savitt A Mische S Li J Marcu KB IKKα, IKKβ, and NEMO/IKKγ are each required for the NF-κB-mediated inflammatory response program J Biol Chem 2002 14 45129 45140 10.1074/jbc.M205165200 12221085 PMC1201411

[B12] Li Q Verma IM NF-κB regulation in the immune system Nat Rev Immunol 2002 14 725 734 10.1038/nri910 12360211

[B13] Sun Shao-Cong Non-canonical NF-κB signaling pathway Cell Res 2011 14 71 85 10.1038/cr.2010.177 21173796 PMC3193406

[B14] Yong Y Choi SW Choi HJ Nam HW Kim JA Jeong DU Kim DY Kim YS Kim DW Exogenous signal-independent nuclear IκB kinase activation triggered by Nkx3.2 enables constitutive nuclear degradation of IκB-α in chondrocytes Mol Cell Biol 2011 14 2802 2816 10.1128/MCB.00253-10 21606193 PMC3133409

[B15] Bayon Y Ortiz MA Lopez-Hernandez FJ Gao F Karin M Pfahl M Piedrafita FJ Inhibition of IκB kinase by a new class of retinoid-related anticancer agents that induce apoptosis Mol Cell Biol 2003 14 1061 1074 10.1128/MCB.23.3.1061-1074.2003 12529410 PMC140693

[B16] El Mansouri FE Chabane N Zayed N Kapoor M Benderdour M Martel-Pelletier J Pelletier JP Duval N Fahmi H Contribution of H3K4 methylation by SET-1A to interleukin-1-induced cyclooxygenase 2 and inducible nitric oxide synthase expression in human osteoarthritis chondrocytes Arthritis Rheum 2011 14 168 179 10.1002/art.27762 20862685

[B17] Srinivasan M Sudheer AR Pillai KR Kumar PR Sudhakaran PR Menon VP Influence of ferulic acid on gamma-radiation induced DNA damage, lipid peroxidation and antioxidant status in primary culture of isolated rat hepatocytes Toxicology 2006 14 249 258 10.1016/j.tox.2006.09.004 17049709

[B18] Yogeeta SK Gnanapragasam A Senthilkumar S Subhashini R Devaki T Synergistic salubrious effect of ferulic acid and ascorbic acid on membrane-bound phosphatases and lysosomal hydrolases during experimental myocardial infarction in rats Life Sci 2006 14 258 263 10.1016/j.lfs.2006.09.012 17045618

[B19] Perluigi M Joshi G Sultana R Calabrese V De Marco C Coccia R Cini C Butterfield DA In vivo protective effects of ferulic acid ethyl ester against amyloid-beta peptide 1-42-induced oxidative stress J Neurosci Res 2006 14 418 426 10.1002/jnr.20879 16634068

[B20] Wenk GL McGann-Gramling K Hauss-Wegrzyniak B Ronchetti D Maucci R Rosi S Gasparini L Ongini E Attenuation of chronic neuroinflammation by a nitric oxide-releasing derivative of the antioxidant ferulic acid J Neurochem 2004 14 484 493 10.1111/j.1471-4159.2004.02359.x 15056291

[B21] Khanduja KL Avti PK Kumar S Mittal N Sohi KK Pathak CM Anti-apoptotic activity of caffeic acid, ellagic acid and ferulic acid in normal human peripheral blood mononuclear cells: a Bcl-2 independent mechanism Biochim Biophys Acta 2006 14 283 289 10.1016/j.bbagen.2005.12.017 16459021

[B22] Shang L Qin J Chen LB Liu BX Jacques M Wang H Effects of sodium ferulate on human osteoarthritic chondrocytes and osteoarthritis in rats Clin Exp Pharmacol Physiol 2009 14 912 918 10.1111/j.1440-1681.2009.05171.x 19298533

[B23] Chakraborti S Mandal M Das S Mandal A Chakraborti T Regulation of matrix metalloproteinases: an overview Mol Cell Biochem 2003 14 269 285 10.1023/A:1026028303196 14619979

[B24] Fernandes JC Martel-Pelletier J Pelletier JP The role of cytokines in osteoarthritis pathophysiology Biorheology 2002 14 237 246 12082286

[B25] Taskiran D Stefanovic-Racic M Georgescu H Evans C Nitric oxide mediates suppression of cartilage proteoglycan synthesis by interleukin-1 Biochem Biophys Res Commun 1994 14 142 148 10.1006/bbrc.1994.1426 7513156

[B26] Bau B Gebhard PM Haag J Knorr T Bartnik E Aigner T Relative messenger RNA expression profiling of collagenases and aggrecanases in human articular chondrocytes in vivo and in vitro Arthritis Rheum 2002 14 2648 2657 10.1002/art.10531 12384923

[B27] Amin AR Abramson SB The role of nitric oxide in articular cartilage breakdown in osteoarthritis Curr Opin Rheumatol 1998 14 263 268 10.1097/00002281-199805000-00018 9608331

[B28] Liacini A Sylvester J Li WQ Zafarullah M Inhibition of interleukin-1-stimulated MAP kinases, activating protein-1 (AP-1) and nuclear factor kappa B (NF-κB) transcription factors down-regulates matrix metalloproteinase gene expression in articular chondrocytes Matrix Biol 2002 14 251 262 10.1016/S0945-053X(02)00007-0 12009331

[B29] Largo R Alvarez-Soria MA Diez-Ortego I Calvo E Sanchez-Pernaute O Egido J Herrero-Beaumont G Glucosamine inhibits IL-1β-induced NFκB activation in human osteoarthritic chondrocytes Osteoarthritis Cartilage 2003 14 290 298 10.1016/S1063-4584(03)00028-1 12681956

[B30] Singh R Ahmed S Islam N Goldberg VM Haqqi TM Epigallocatechin-3-gallate inhibits interleukin-1β-induced expression of nitric oxide synthase and production of nitric oxide in human chondrocytes: suppression of nuclear factor κB activation by degradation of the inhibitor of nuclear factor κB Arthritis Rheum 2002 14 2079 2086 10.1002/art.10443 12209512

[B31] Liacini A Sylvester J Li WQ Huang W Dehnade F Ahmad M Zafarullah M Induction of matrix metalloproteinase-13 gene expression by TNF-α is mediated by MAP kinases, AP-1, and NF-κB transcription factors in articular chondrocytes Exp Cell Res 2003 14 208 217 10.1016/S0014-4827(03)00180-0 12878172

[B32] Shakibaei M John T Schulze-Tanzil G Lehmann I Mobasheri A Suppression of NF-κB activation by curcumin leads to inhibition of expression of cyclo-oxygenase-2 and matrix metalloproteinase-9 in human articular chondrocytes: implications for the treatment of osteoarthritis Biochem Pharmacol 2007 14 1434 1445 10.1016/j.bcp.2007.01.005 17291458

[B33] Carames B Lopez-Armada MJ Cillero-Pastor B Lires-Dean M Vaamonde C Galdo F Blanco FJ Differential effects of tumor necrosis factor-alpha and interleukin-1β on cell death in human articular chondrocytes Osteoarthritis Cartilage 2008 14 715 722 10.1016/j.joca.2007.10.006 18054255

[B34] Li H Lin X Positive and negative signaling components involved in TNFα-induced NF-κB activation Cytokine 2008 14 1 8 10.1016/j.cyto.2007.09.016 18068998

[B35] Aizawa T Kon T Einhorn TA Gerstenfeld LC Induction of apoptosis in chondrocytes by tumor necrosis factor-alpha J Orthop Res 2001 14 785 796 10.1016/S0736-0266(00)00078-4 11562122

[B36] Basak S Shih VF Hoffmann A Generation and activation of multiple dimeric transcription factors within the NF-κB signaling system Mol Cell Biol 2008 14 3139 3150 10.1128/MCB.01469-07 18299388 PMC2423155

[B37] Roman-Blas JA Jimenez SA NF-κB as a potential therapeutic target in osteoarthritis and rheumatoid arthritis Osteoarthritis Cartilage 2006 14 839 848 10.1016/j.joca.2006.04.008 16730463

[B38] Carlsen H Alexander G Austenaa LM Ebihara K Blomhoff R Molecular imaging of the transcription factor NF-κB, a primary regulator of stress response Mutat Res 2004 14 199 211 10.1016/j.mrfmmm.2004.02.024 15225593

[B39] Sun SC Ganchi PA Ballard DW Greene WC NF-κB controls expression of inhibitor IκBα: evidence for an inducible autoregulatory pathway Science 1993 14 1912 1915 10.1126/science.8096091 8096091

[B40] Johnson C Van Antwerp D Hope TJ An N-terminal nuclear export signal is required for the nucleocytoplasmic shuttling of IκBα Embo J 1999 14 6682 6693 10.1093/emboj/18.23.6682 10581242 PMC1171731

[B41] Wang BH Ou-Yang JP Pharmacological actions of sodium ferulate in cardiovascular system Cardiovasc Drug Rev 2005 14 161 172 16007232 10.1111/j.1527-3466.2005.tb00163.x

[B42] Sun YM Lou JT Huang GQ Clinical study on sodium ferulate for intracerebral hemorrhage in early stage Zhongguo Zhong Yao Za Zhi 2008 14 2545 2548 19149269

[B43] Yin H Yan X Yang KH Systematic review on sodium ferulate for treatment of diabetic nephropathy Zhongguo Zhong Xi Yi Jie He Za Zhi 2009 14 970 974 20329603

[B44] Barone Eugenio Calabrese Vittorio Mancuso Cesare Ferulic acid and its therapeutic potential as a hormetin for age-related diseases Biogerontology 2009 14 97 108 10.1007/s10522-008-9160-8 18651237

